# Supplementary material for: End Sequence Analysis Toolkit (ESAT) expands the extractable information from single-cell RNA-seq data
Source: Genome Res. 2016 Oct;26(10):1397–410. doi: 10.1101/gr.207902.116 (PMC5052061; doi:10.1101/gr.207902.116)
Supplement: Supplemental Material [file supp_gr.207902.116_Supp_Materials.tar.gz › ESAT-esat_v0.1/doc/html/classbroad_1_1core_1_1primer3_1_1_compute_m_i_r_score.html]

ESAT: broad.core.primer3.ComputeMIRScore Class Reference


ESAT


- Main Page
- Related Pages
- Packages
- Classes
- Files

- Class List
- Class Index
- Class Hierarchy
- Class Members

All Classes Namespaces Files Functions Variables Enumerator Pages

- broad
- core
- primer3
- ComputeMIRScore

Static Public Member Functions |
List of all members

broad.core.primer3.ComputeMIRScore Class Reference

Collaboration diagram for broad.core.primer3.ComputeMIRScore:

[legend]

|  |  |
| --- | --- |
| Static Public Member Functions | |
| static double | computeMIRScore (String sequence, File mirSeedLookup, File miRNALookup) throws IOException |
|  | |
| static double | computeMIRScore (String sequence, Map< String, Double > mirLookup, Set< String > lookup2, Set< String > rcLookup2) |
|  | |
| static double | computeMIRScore (String sequence) throws IOException |
|  | |
| static Set | parse (File file, boolean rc) throws IOException |
|  | |
| static String | reverseComplement (String seq) |
|  | |
| static String | reverse (String seq) |
|  | |
| static String | complement (String seq) |
|  | |
| static void | main (String[] args) throws IOException |
|  | |

## Member Function Documentation

|  |  |  |  |  |  |
| --- | --- | --- | --- | --- | --- |
| static String broad.core.primer3.ComputeMIRScore.complement | ( | String | *seq* | ) |  |

static

|  |  |  |  |
| --- | --- | --- | --- |
| static double broad.core.primer3.ComputeMIRScore.computeMIRScore | ( | String | *sequence*, |
|  |  | File | *mirSeedLookup*, |
|  |  | File | *miRNALookup* |
|  | ) |  | throws IOException |

static

Here is the call graph for this function:

Here is the caller graph for this function:

|  |  |  |  |
| --- | --- | --- | --- |
| static double broad.core.primer3.ComputeMIRScore.computeMIRScore | ( | String | *sequence*, |
|  |  | Map< String, Double > | *mirLookup*, |
|  |  | Set< String > | *lookup2*, |
|  |  | Set< String > | *rcLookup2* |
|  | ) |  |  |

static

|  |  |  |  |  |  |
| --- | --- | --- | --- | --- | --- |
| static double broad.core.primer3.ComputeMIRScore.computeMIRScore | ( | String | *sequence* | ) | throws IOException |

static

Here is the call graph for this function:

|  |  |  |  |  |  |
| --- | --- | --- | --- | --- | --- |
| static void broad.core.primer3.ComputeMIRScore.main | ( | String[] | *args* | ) | throws IOException |

static

|  |  |  |  |
| --- | --- | --- | --- |
| static Set broad.core.primer3.ComputeMIRScore.parse | ( | File | *file*, |
|  |  | boolean | *rc* |
|  | ) |  | throws IOException |

static

Here is the call graph for this function:

Here is the caller graph for this function:

|  |  |  |  |  |  |
| --- | --- | --- | --- | --- | --- |
| static String broad.core.primer3.ComputeMIRScore.reverse | ( | String | *seq* | ) |  |

static

Here is the caller graph for this function:

|  |  |  |  |  |  |
| --- | --- | --- | --- | --- | --- |
| static String broad.core.primer3.ComputeMIRScore.reverseComplement | ( | String | *seq* | ) |  |

static

Here is the call graph for this function:

Here is the caller graph for this function:

---

The documentation for this class was generated from the following file:

- java/broad/core/primer3/ComputeMIRScore.java


---

Generated on Fri Aug 15 2014 15:10:55 for ESAT by  

 1.8.7
